# Evaluation of sonication on stability-indicating properties of optimized pilocarpine hydrochloride-loaded niosomes in ocular drug delivery

**DOI:** 10.1007/s40204-021-00164-5

**Published:** 2021-09-22

**Authors:** Kruga Owodeha-Ashaka, Margaret O. Ilomuanya, Affiong Iyire

**Affiliations:** 1 Aston Pharmacy School, College of Life and Health Sciences, Aston University, Birmingham, B4 7ET UK; 2 Department of Pharmaceutics and Pharmaceutical Technology, Faculty of Pharmacy, University of Lagos, Yaba, Lagos State Nigeria

**Keywords:** Niosomes, Sonication, Stability, Non-ionic surfactants

## Abstract

Niosomes are increasingly explored for enhancing drug penetration and retention in ocular tissues for both posterior and anterior eye delivery. They have been employed in encapsulating both hydrophilic and hydrophobic drugs, but their use is still plagued with challenges of stability and poor entrapment efficiency particularly with hydrophilic drugs. As a result, focus is on understanding the parameters that affect their stability and their optimization for improved results. Pilocarpine hydrochloride (HCl), a hydrophilic drug is used in the management of intraocular pressure in glaucoma. We aimed at optimizing pilocarpine HCl niosomes and evaluating the effect of sonication on its stability-indicating properties such as particle size, polydispersity index (PDI), zeta potential and entrapment efficiency. Pilocarpine niosomes were prepared by ether injection method. Composition concentrations were varied and the effects of these variations on niosomal properties were evaluated. The effects of sonication on niosomes were determined by sonicating optimized drug-loaded formulations for 30 min and 60 min. Tween 60 was confirmed to be more suitable over Span 60 for encapsulating hydrophilic drugs, resulting in the highest entrapment efficiency (EE) and better polydispersity and particle size indices. Optimum sonication duration as a process variable was determined to be 30 min which increased EE from 24.5% to 42% and zeta potential from (−)14.39 ± 8.55 mV to (−)18.92 ± 7.53 mV. In addition to selecting the appropriate surfactants and varying product composition concentrations, optimizing sonication parameters can be used to fine-tune niosomal properties to those most desirable for extended eye retainment and maintenance of long term stability.

## Introduction

Optimum drug delivery to the eye is especially difficult as the eye possesses intrinsic anatomical and physiological properties that pose barriers. There is therefore the problem of poor bioavailability and an associated need for frequent administrations to achieve and maintain optimum ocular concentrations. Pilocarpine hydrochloride is used for managing intraocular pressure (IOP) in the treatment of glaucoma. It is a miotic drug that acts as a muscarinic agonist, causing ciliary muscle contraction that opens up the trabecular meshwork which allows aqueous humour drainage and a resultant reduction in IOP (Jain and Verma 2020). It is hydrophilic in nature and its use as a conventional eye drop is faced with the challenges highlighted prior, leading to short retention times, low bioavailability and reduced efficacy (Keipert et al. 1996). It is currently available in other dosage forms as oral tablets, ocular inserts, which are associated with limitations such as poor bioavailability and invasiveness due to inserts.

In solving the above problems, many different formulation strategies are being explored to improve drug solubility, precorneal absorption and retention time in the eye. Nanomicelles, microemulsions, in situ gels and liposomes are some nanotechnology-based systems that have been investigated for pilocarpine delivery (Anumolu et al. 2009; Cholkar et al. 2013; Naveh et al. 1994). Of note are niosomes which serve as nanocarriers and are fundamentally composed of amphiphilic non-ionic surfactants with a polar head and a non-polar tail, lipids like cholesterol, a hydration medium and other additives (Bhardwaj et al. 2020). They are formed as a result of partitioning upon tensile interactions of the aqueous solution and the lipophilic tails of the amphiphilic non-ionic surfactants, causing the tails to associate and leaving the polar hydrophilic heads pointing outwards in contact with the aqueous phase (Seleci et al. 2016). It is able to achieve localized controlled and sustained release in addition to protection of the drug from degradation by metabolic enzymes resident in the eye (Sahoo et al. 2014). Because it is a lipid vesicular system, absorption is increased and reduced systemic drainage results in longer drug contact time, and therefore bioavailability is improved compared to conventional drug solutions (Sahoo et al. 2014).

Different classes of surfactants have been used in the preparation of niosomes. Their properties—size, structure, hydrophilic/lipophilic balance (HLB) value and physical state, and phase transition temperature (Tc)—as well as the concentrations in which they are used, influence vesicle size, polydispersity index (PDI) encapsulation efficiency, charge and stability (Bnyan et al. 2018). Cholesterol interacts with the hydrophobic alkyl end of the surfactant producing an increase in vesicle transition temperature and alteration of bilayer fluidity which stabilizes the membrane (Chen et al. 2019). Cholesterol affects the vesicle’s permeability and drug release, membrane rigidity, encapsulation efficiency, toxicity and stability (Bhardwaj et al. 2020).

Size reduction is beneficial for preventing ocular irritation and inflammation, enhancing pharmacokinetics/drug biodistribution by increased surface area to volume ratio, promoting intracellular delivery and increasing retention time (Prabhu et al. 2010; Nowroozi et al. 2018). Several methods for modifying size reduction to meet desired parameters exist. They include sonication (bath and probe), microfluidization, high-pressure homogenization and extrusion through filters (Uchegbu and Vyas 1998). Sonication is the application of sound energy to a liquid containing particles and has been known for its effects on lipid membranes to produce nano-sized vesicles (Essa 2010). Frequencies greater than 20 Hz are usually used so it is referred to “ultrasonication”. It is a commonly used method for effectual creation of smaller unilamellar vesicles from larger multilamellar vesicles in a lamellar dispersion (Zasadzinski et al.^2011^).

A major challenge that continually hinders progress in the clinical application of niosomes is the issue of their stability. As such, extensive research has gone into investigating formulation and process parameters that influence stability, evaluating niosomal characteristics such as particle size, polydispersity index, zeta potential, and entrapment efficiency, which are often indicative of the relatively unchanged nature of a formulation (Seleci et al. 2016; Chen et al. 2019). Formulations containing charge inducers that ensure adequate electrostatic repulsion between vesicles to prevent aggregation, have shown promise (Bhardwaj et al. 2020). Since the composition and manufacturing process affect product properties, a successful optimization of these parameters give great promise for finally arriving at niosomal formulations that maintain optimum characteristics throughout the cycle of preparation, movement, storage and final use. We will attempt to shed more light on how sonication in the formulation process could play an important role in optimizing pilocarpine hydrochloride niosomal properties that affect their bioavailability and stability. In this work, we formulate, optimize and evaluate the characteristics of pilocarpine hydrochloride-loaded niosomes prepared by ether injection method and determine the effect of sonication time on the above properties.

## Materials

Methanol 99.8% (Fisher Scientific, UK), pilocarpine hydrochloride (HCl) (Sigma Aldrich, Brazil), deionized water, sodium chloride (Sigma Aldrich, Switzerland), potassium chloride (Sigma Aldrich, Spain), sodium hydrogen dibasic phosphate (Sigma Aldrich), potassium dihydrogen phosphate (Fisher Scientific, UK), Tween 60 from (CRODA, UK), Span 60 (CRODA, UK), cholesterol (Sigma Aldrich, USA), diethyl ether 99.7% (Sigma Aldrich, Germany), ethanol 99.8% (Fisher Scientific, UK). All reagents used were of analytical grade.

## Methods

### Reverse-phase HPLC method validation

An Agilent Technologies 1220 Infinity II LC system was used in pilocarpine HCl quantification based on a method developed by Fan et al. (1996) and outlined by El Deeb et al. (2006). The mobile phase was a mixture of solution A, containing 13.5 mL phosphoric acid, 3 mL trimethylamine and 983.5 mL deionized water, and solution B as methanol in a ratio of 98:2. A standard Gemini^®^ 5 μm C18 110A LC column 150 × 4.6 mm was used under ambient experimental conditions (18–21 °C). Flow rate was set at 1.5 mL/min and injection volume was 20μL. The UV absorbance wavelength of pilocarpine hydrochloride was determined to be 215 nm and as such, UV detection was done at this wavelength. Validation was carried out according to ICH guidelines Q2R1 (2005) over a linearity range of 7.8125–500 µg/mL with coefficient of variation *r*^2^ = 0.9999. The method was precise with repeatability giving 1.1% RSD; mean % recovery ranging from 78.38 to 103.93%; and limits of detection and quantification at 0.158 μg/mL and 0.528 μg/mL respectively. All but one of the mean recovery RSD% values were under the acceptable 15% upper limit for pharmaceutical analysis (Iyire et al. 2018). Recovery at the lowest concentration in the range gave the lowest recovery, informing the use of the indirect method for quantifying the amount of pilocarpine hydrochloride entrapped in the niosomes.

### Compatibility studies

Compatibility studies between pilocarpine hydrochloride and cholesterol were previously done in the lab using FTIR and DSC and the data are already published by Alyami et al. (2020). DSC thermograms and FTIR spectra obtained established the absence of drug-excipient interactions or potential for incompatibilities in the formulation.

### Preparation of niosomes

Niosomes were prepared by ether injection method as described by Ravalika and Krishna (2017) with slight modifications. Composition ratios are shown in Table 1. Ethanol was used instead of methanol and the drug was dissolved in the aqueous phase (phosphate buffer) as it was insoluble in the organic solvents at the quantities used. Briefly, the surfactant(s) together with cholesterol were accurately weighed into a beaker. A mixture of 2 mL ethanol and 6 mL diethyl ether was added to the beaker and gently agitated to facilitate dissolution. The beaker was covered with parafilm to reduce solvent evaporation to the barest minimum. Upon complete dissolution, the solution was withdrawn using a syringe fitted with a 23G needle. To a sample vial containing 40 mg pilocarpine HCl dissolved in 10 mL phosphate buffer previously warmed to and maintained at 60–62 °C in a water bath, the solution was slowly injected at 0.8 mL/min while magnetically stirring at 100 rpm. Care was taken to ensure the solution was injected into the phosphate buffer and not above it. The resulting suspension was continually stirred for 45 min to allow for solvent evaporation and a well dispersed suspension.

**Table 1:** Niosomal formulation compositions showing concentration of drug, surfactant(s) and cholesterol used

Formulation	Tween 60(mg)	Span 60(mg)	Cholesterol(mg)	Pilocarpine HCl (mg)
K1	100	–	100	40
K2	–	100	100	40
K3	50	50	100	40
K4	50	–	100	40
K5	–	50	100	40
K6	25	25	100	40
K7	100	–	50	40
K8	–	100	50	40
K9	50	50	50	40

### Characterisation of niosomes

#### Visual inspection and morphology

The formulated suspensions were inspected for their physical appearance and colour. Redispersed niosomal suspension was viewed on a glass slide under a Carl Zeiss Analytical Microscope (Germany) using Axiovision software through lenses × 10, × 40 and × 100. The viewing was adjusted to acquire as clear an image as possible which was captured by an attached camera.

#### Particle size, polydispersity index (PDI) and zeta potential

These parameters were evaluated according to a procedure adopted from Sankhyan and Pawar (2013). Particle size and PDI were determined using dynamic light scattering (DLS) technique in a Brookhaven Zetasizer, model Nanobrook 90plus zeta (USA) with BIC particle solutions software. Electrophoretic light scattering (ELS) technique in the same zetasizer was used to determine zeta potential at a temperature of 25 °C.

#### Entrapment efficiency

This was carried out as described by Verma et al. (2019) with slight modifications. 1 mL of the niosomal suspension was measured into a 1.5 mL capacity Eppendorf tube and placed in a cooling centrifuge, Prism R model from Labnet International Inc. (USA), ensuring proper centrifuge balance. The centrifugation parameters were set at 4 °C, 11,000 × g force and run time of 1 h. After centrifugation, the supernatant was carefully drawn up into appropriately labelled containers and the separated pellets were washed. Washing was done by adding 1 mL of pH 7.4 phosphate buffer to the solute in the tube and mixing. The mixture was centrifuged for another 1 h and all the supernatant was carefully collected and stored in foil-wrapped sample vials to protect pilocarpine HCl from light degradation.

The quantity of total supernatant recovered was determined by measurement after which the supernatant was filtered and analyzed using an Agilent Technologies 1220 Infinity II LC HPLC system to determine the amount of free unencapsulated drug. Indirect determination of EE was carried using Eq. (1):

1 $$\frac{\mathrm{Total drug}-\mathrm{Free drug}}{\mathrm{Total drug}} \times 100$$

### Statistical analysis

GraphPad Prism software, version 8.4.3 from GraphPad (USA) was used in the statistical analysis of results obtained. Data was compared using one-way analysis of variance (ANOVA) and two-way ANOVA followed by Tukey or Sidak post-test as indicated. Differences with *p* < 0.05 were considered significant. All particle sizes, PDI and zeta potentials were taken in repetitions (*n* = 6) and were presented as mean ± standard deviation.

## Results

### Visual inspection and morphology

**Fig. 1:**
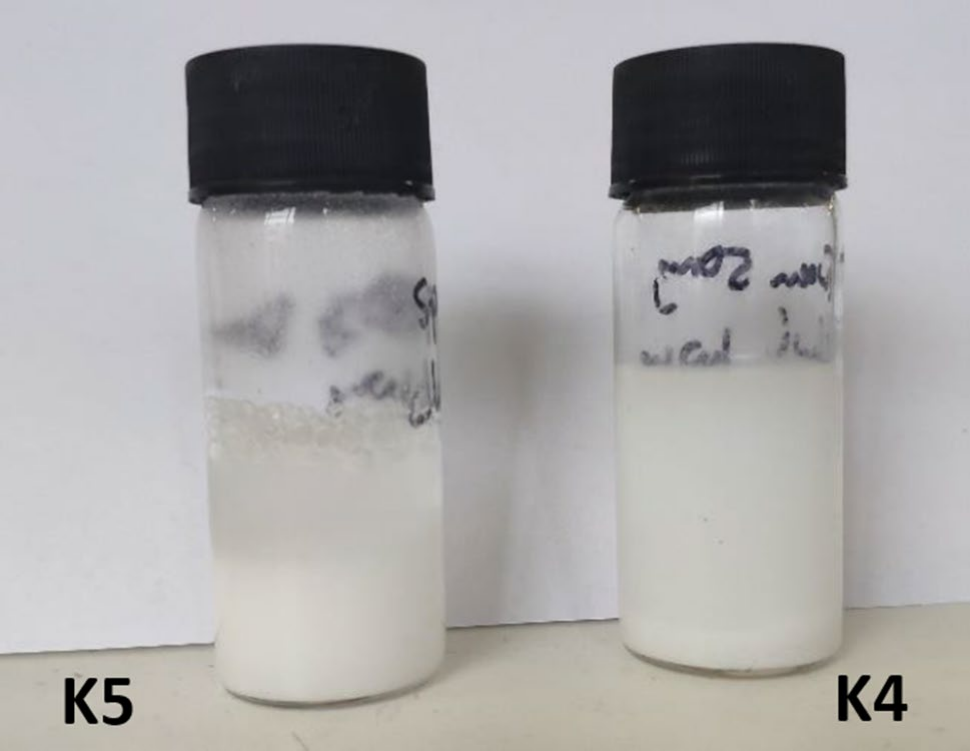
Visual observation of niosomal formulations K5 (0.5:1, Span 60:cholesterol) and K4 (0.5:1, Tween 60:cholesterol) on standing

The formulated niosomal suspensions were milky/cloudy with a well dispersed fluid consistency. The formulations containing Span 60 were observed to be cloudier than those with Tween 60. On standing, a greater separation of the Span 60 niosomal suspension was observed, with the settling of vesicles at the bottom of the vial and a clear aqueous phase above. Tween 60 niosomes did not show the same degree of separation as the supernatant above remained significantly cloudy (Fig. 1).

**Fig. 2:**
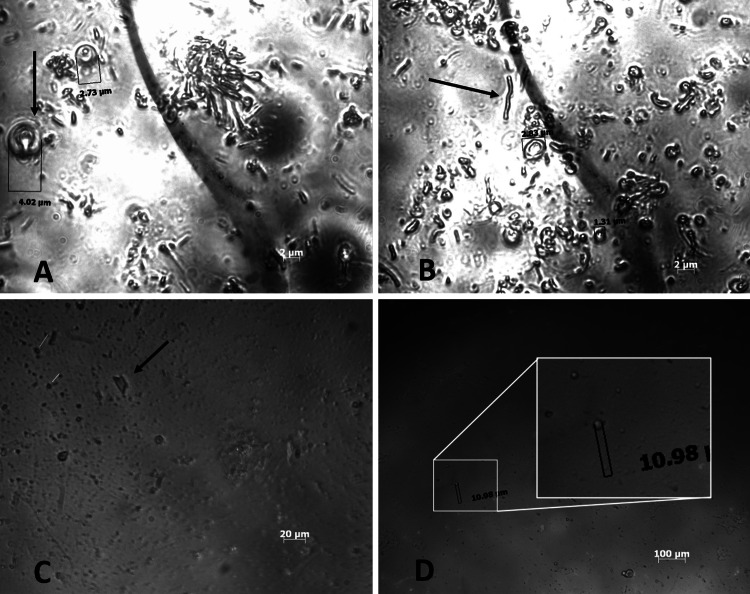
Micrographs of drug loaded formulations **A** (K8) × 100 showing malformed niosomes, **B** (K2) drug-loaded × 100 showing tubules, **C** (K4) blank × 40 showing crystal structure, and **D** (K4) blank × 10 showing well formed niosomes

Nisosomes formed were spherical (Fig. 2D), corresponding with other studies in which Span 60 and Tween 60 were used (Junyaprasert et al. 2012). Vesicles were unilamellar as is characteristic of niosomes formed by ether injection method. Span 60 niosomes were seen as ‘densely’ packed aggregates with a few disperse vesicles and having a greater proportion of visibly larger lamellar vesicles compared with Tween 60. For some Span 60 containing formulations as seen in Fig. 2A, incompletely formed vesicles were observed.

Elongated vesicles and tubules were observed in some Span 60 formulations, Fig. 2B, as has also been reported by Barakat et al. (2014), Marwa et al. (2013) and Rangasamy et al. (2008). The formulations containing Tween 60 were composed of smaller spherical disperse unilamellar vesicles, with some showing small crystal-like structures (Fig. 2C).

### Particle size and polydispersity index

Tween 60 produced particle sizes generally smaller than those of Span 60. K2 had the largest size at 1,229.87 ± 277.24 nm and was significantly larger than other formulations, *p* < 0.0001 (Table 2). The corresponding K1 formulation containing Tween 60 had smaller sizes of 516.69 ± 30.22 nm. K4 (Tween 60:cholesterol, 0.5:1) was the smallest in size at 318.90 ± 26.97 nm, which was significantly different compared with other formulations *p* ≤ 0.0220.

**Table 2:** Summary of particle size analysis and PDI for blank niosomes

Blank formulation	Particle size (nm) (mean ± SD)	Polydispersity index (mean ± SD)
K1; Tween 1: Chol 1	516.69 ± 30.22	0.21 ± 0.14
K2; Span 1: Chol 1	1229.87 ± 277.24	0.16 ± 0.12
K3; Tween 0.5/Span 0.5: Chol 1	556.56 ± 76.73	0.23 ± 0.21
K4; Tween 0.5: Chol 1	318.90 ± 26.97	0.36 ± 0.08
K5; Span 0.5: Chol 1	819.87 ± 232.99	0.51 ± 0.69
K6; Tween 0.25/Span 0.25: Chol 1	730.73 ± 91.69	0.47 ± 0.18
K7; Tween 1: Chol 0.5	484.49 ± 15.00	0.28 ± 0.18
K8; Span 1: Chol 0.5	535.15 ± 125.84	2.52 ± 1.88****
K9; Tween 0.5/Span 0.5: Chol 0.5	844.22 ± 112.79	0.24 ± 0.15

All formulations gave homogenous dispersions except K8 (Span 60:cholesterol, 1:0.5) with a PDI greater than 0.7 showing statistically significant differences (*p* < 0.0001) by one-way ANOVA. Data is presented as mean of six determinations ± standard deviation

* 0.05; 0.01 to 0.05 - Significant

** ≤ 0.01; 0.001 to 0.01 – Very significant

*** ≤ 0.001; 0.0001 to 0.001 – Extremely significant

**** ≤ 0.0001 – Extremely significant

Increased surfactant concentrations gave mostly larger sized vesicles for both Span 60 and Tween 60 niosomes, with Span 60’s influence being more significant, *p* < 0.0001 (Table 2). Similarly, for corresponding concentrations of both surfactants, lower cholesterol concentrations in the composition ratio produced smaller sized niosomes. Upon drug loading, formulations containing Span 60 registered increase in particle size (*p* ˂0.0001, two-way ANOVA, Sidak’s post-test), presented in Table 3.

**Table 3:** Summary of particle size analysis, PDI, zeta potential (data is presented as mean ± SD for 6 determinations) and entrapment efficiency for pilocarpine HCl-loaded niosomes. K2 (Span 60:cholesterol, 1:1) showed statistically significant differences (****; *p* < 0.0001, one-way ANOVA) in zeta potential compared with other formulations except K8 (Span 60:cholesterol, 1:0.5)

Drug loaded Formulation	Particle size (mean ± SD)	Polydispersity index (mean ± SD)	Zeta Potential (mV) (mean ± SD)	% EE using free drug
K1; Tween 1: Chol 1	294.37 ± 14.73	0.09 ± 0.07	− 24.16 ± 3.38	34.0
K2; Span 1: Chol 1	1124.61 ± 389.28	2.43 ± 1.73***	− 54.66 ± 4.26****	21.9
K3; Tween 0.5/Span 0.5: Chol 1	520.76 ± 37.01	0.25 ± 0.27	− 33.13 ± 10.71	26.5
K4; Tween 0.5: Chol 1	335.42 ± 18.23	0.17 ± 0.06	− 10.61 ± 3.59	20.6
K5; Span 0.5: Chol 1	1644.92 ± 254.23	1.69 ± 1.20	− 26.68 ± 10.93	25.1
K6; Tween 0.25/Span 0.25: Chol 1	713.77 ± 94.11	0.28 ± 0.15	− 22.68 ± 8.20	24.2
K7; Tween 1: Chol 0.5	433.41 ± 41.58	0.07 ± 0.03	− 13.06 ± 2.66	24.2
K8; Span 1: Chol 0.5	1007.81 ± 178.95	0.76 ± 1.00	− 41.36 ± 5.76	18.3
K9; Tween 0.5/Span 0.5: Chol 0.5	1054.69 ± 157.68	0.25 ± 0.09	− 26.19 ± 8.37	23.0

PDI for K2 was also significantly larger than other formulations except K5 (Span 60:cholesterol, 0.5:1) (*p* < 0.0007)

The PDI for all the blank formulations ranged from 0.16 to as high as 2.52 (Table 2), although this high value was seen only in formulation K8. Other formulations had acceptable PDIs from 0.16 to 0.51 indicating a homogenous dispersion (Rehman et al. 2018). K8 with 2.52 translated to a dispersion lacking homogeneity as seen by the distribution of particle sizes, some of which are up to twice the size of the lowest size recorded. Tween 60 formulations had the lowest values and it can be said that smaller particle sizes gave better PDI (Kaur et al. 2016). For both the Tween 60 and Span 60 singly composed formulations, higher surfactant concentrations gave lower PDIs, an observation in line with that made by Sharma et al. (2016). By an analysis using one-way ANOVA followed by Tukey post-test, K8 showed significant differences (*p* ≤ 0.0002) from the other formulations. Following drug loading, Span-only niosomes were seen to have generally higher PDIs, only running as low as 0.76 which is typically not considered desirable (Table 3).

### Zeta potential

In this study, Tween 60 containing formulations had lower values (p < 0.0001, one-way ANOVA followed by Tukey post-test) than their counterpart Span 60 vesicles. As shown in Table 3, K2 (Span 60:cholesterol, 1:1) recorded the highest zeta potential of − 54.66 ± 4.26 mV, while K8 (Span 60:cholesterol, 1:0.5) had a slightly lower value of − 41.36 ± 5.76 mV and K5 (Span 60:cholesterol, 0.5:1) gave − 26.68 ± 10.93 mV, just about half that of K2's. This same trend was observed in the Tween 60 containing formulations with K1 giving zeta values of − 24.16 ± 3.38 mV, K7 giving − 13.06 ± 2.66 mV, and K4 giving − 10.61 ± 3.59 mV.

Zeta potential was seen to increase (more negative) with an increase in surfactant ratio in Span 60 formulations from − 22.68 ± 8.2 mV in K5 to − 54.66 ± 4.26 mV in K2. Tween 60 as well as the co-surfactant formulations followed the same trend. For the surfactant combinations, K3 with equal surfactant:cholesterol ratio gave the highest zeta value of − 33.13 ± 10.71 mV, while K9 with half the cholesterol concentration giving a slightly lower − 26.19 ± 8.37 mV and K6 with half the surfactant concentration giving the lowest value at − 22.68 ± 8.2 mV. These results are a seemingly good demonstration of the effects of different surfactant types as has been described by other researchers (Bnyan et al. 2018). Summarily, K2 showed statistically significant differences in zeta potential value (*p* ≤ 0.001, one-way ANOVA followed by Tukey test) compared to other formulations except K8 where there was no significant difference (*p* > 0.05), which is understandable as it had the same Span 60 concentration as K2.

### Entrapment efficiency

K1 containing equal Tween 60 and cholesterol gave the highest EE% of 34% compared with K2 having Span 60 which gave 21.9%, Table 3. EE was seen to increase for Tween 60 formulations from 20.6% in K4 to 34% in K1 as surfactant concentration increased, translating to a larger aqueous space so more drug uptake. The co-surfactant formulations followed the same trend as K3 with a higher total surfactant concentration produced a slightly higher EE, 26.5% than K6, 24.2%.

Comparing K1 and K7, K2 and K8 and K3 and K9 to demonstrate the effect of cholesterol concentration, the higher cholesterol containing formulations all gave higher EE. With half the cholesterol concentration, formulations K7 with Tween 60, K9 combining Span 60 and Tween 60 and K8 with Span 60 gave EE of 24.2%, 23% and 18.3% respectively. The effect of increase in cholesterol concentration was clearly observed when comparing K1 and K7, where K1 having the higher concentration gave an EE of 34% and K7 gave 24.2%.

In these experiments, as with results obtained by Palozza et al. in (2006) using β-carotene niosomes, EE did not correlate with size particularly with Tween 60 formulations as K1 with the smallest size had a higher EE than K2 with a much larger size. However, the size and EE for K2 and K5 containing Span 60 did  correlate, with the larger K5 showing the higher EE.

### Effects of sonication on niosomal properties

K7 containing Tween 60 was chosen to evaluate the effect of sonication on the niosomal dispersion because it gave good cumulative properties in terms of particle size, polydispersity index and zeta potential. For good comparability, K8 containing the same concentrations of cholesterol and surfactant, in this case Span 60 was also chosen. This was to determine any possible influence the type of surfactant would have on the effect of sonication.

Sonication was carried out using a Fisherbrand bath sonicator and the sample vial was placed in an ice bath to maintain a cool temperature as the process usually results in generation of heat. This process was described by Mavaddati et al. (2015) as they investigated its effects on the physical character of niosomes of dexamethasone. It is based on the principle of generation and oscillation of formed bubbles i.e., cavitation in liquids by ultrasound mechanical waves. Application of a frequency of resonant size leads to the nonlinear oscillation and eventual collapse of bubbles with sizes near those of the frequency applied. The collapse results in generation of extremely high temperatures, shock waves and high pressures. Larger vesicles are then randomly but uniformly broken down by the ultrasonic high energy to small discoid fragments which fold up to form thermodynamically stable vesicles.

### Visual inspection and morphology

For both formulations, the separation of solvent and solute phases was more evident and marked by a sediment below and a clear phosphate buffer solution above. Large unilamellar vesicles were produced for both formulations as earlier noted. Especially for K8 containing Span 60, longer sonication time and the associated particle size reduction led to the vesicles becoming more discrete. K7 with Tween 60 also behaved in this way but to a lesser degree that could be observed. De et al. (2018) made similar observations after probe sonicating niosomes of temozolomide.

### Particle size and polydispersity index (PDI)

Particle sizes generally reduced after sonication with a more significant decrease after 60 min for both Span 60 and Tween 60 formulations (Table 4). With no sonication, K7a containing Tween 60 had an average size of 338.74 ± 14.37 nm only reducing slightly after 30 min of sonication (K7b) to 334.94 ± 19.80 nm. After 60 min (K7c), there was a more noticeable decrease in particle size to 270.35 ± 17.21 nm. For K8a, with no sonication, average size was 1,900.54 ± 610 nm. After 30 min of sonication (K8b), there was a decrease to 1,308.55 ± 310.90 nm and sonication for 60 min (K8c) resulted in a 75.6% decrease in size to 462.89 ± 42.47 nm. The difference in size of K8a and K8b from the other formulations and between themselves was statistically significant (*p* < 0.0001, by two-way ANOVA followed by Tukey test). There was no statistically significant difference between K8c and K7a, b and c (*p* > 0.05) as the sizes were close in range as shown in Table 4.

**Table 4:** Effects of sonication on niosomal character from 0-60 min

Formulation	Particle size (nm) (mean ± SD)	Polydispersity index (mean ± SD)	Zeta potential (mV) (mean ± SD)	%EE using free drug
K7a No sonication	338.74 ± 14.37	0.18 ± 0.05	− 14.39 ± 8.55	26.5
K7b 30 min	334.94 ± 19.80	0.18 ± 0.02	− 18.92 ± 7.53	42.7
K7c 60 min	270.35 ± 17.21	0.20 ± 0.11	− 17.78 ± 7.62	35.8
K8a No sonication	1900.54 ± 610.39****	0.41 ± 0.52	− 33.34 ± 12.91	23.0
K8b 30 min	1308.55 ± 310.90****	0.30 ± 0.07	− 43.94 ± 12.68	20.6
K8c 60 min	462.89 ± 42.47	0.27 ± 0.08	− 51.49 ± 6.10	21.6

Data presented as mean ± SD where indicated are for 6 determinations. K8a and b showed statistically significant differences (****; *p* < 0.0001, two-way ANOVA)

As shown in Table 4 above, there was not much change in the PDI of the K7 through the sonication process. For K8 containing Span 60, the PDI was seen to consistently reduce in value from 0.41 with no sonication (K8a) to 0.27 60 min after sonication (K8c). Although changes in PDI values can be seen, they were not considered statistically significant (*p* > 0.05, one-way ANOVA followed by Tukey test).

### Zeta potential

As previously determined in non-sonicated drug-loaded vesicles, the Span 60 containing formulation had a higher (more negative) zeta potential than its Tween 60 counterpart. For K8, zeta potential consistently increased from -33.34 mV at 0 min of sonication to − 43.94 mV at 30 min to − 51.49 at 60 min, Table 4. Tween 60 containing K7 showed an increase in zeta potential from − 14.39 mV prior to sonicating to − 18.92 mV after 30 min. It remained significantly unchanged after 60 min at a potential of − 17.78 mV.

### Entrapment efficiency

For K7, EE was seen to increase from 26.5% at no sonication to 42.7% after sonicating for 30 min, Table 4. On further sonication up to 60 min as seen with K7c, EE reduced to 35.8%. For K8 a decrease from 23% before sonication to 20% after 30 min of sonication, followed by a statistically insignificant increase to 21.6% after 60 min was observed.

## Discussion

### Effect of Surfactant and cholesterol concentration on niosomal properties

The use of pilocarpine HCl is a long-standing therapeutic strategy in the management of open-angle glaucoma and acute angle-closure glaucoma. As a hydrophilic drug, the corneal epithelium being lipophilic is the major barrier to its permeation after topical administration, retarding the passage of up to 90% of administered drug (Loftsson et al. 2008). It is still most widely available as conventional ophthalmic solutions for topical administration in drops, as well as suspensions and gel-based formulations. The fundamental challenges with these dosage forms are the associated poor ocular retention, limited precorneal absorption and loss due to nasolacrimal drainage, necessitating the need for frequent administrations which negatively affect patient compliance and ultimately, treatment outcomes (Gaudana et al. 2009). Niosomes have shown great potential for addressing these challenges in ocular delivery due to the nature of their structure and composition which offers the advantage of being biodegradable, biocompatible and non-immunogenic (Sahoo et al. 2014).

The cloudy appearance of formulated niosomes was typical and consistent with reports by Shah et al. (2020). Clear differences in the degree of separation on standing can be attributed to the intrinsic physical character of the surfactants. Span 60 being more hydrophobic (HLB value of 4.7) than Tween 60 (HLB value of 14.9) would exhibit less interaction with the aqueous phase. It is well known that the method of preparation influences the type of resulting niosomes. The unilamellar vesicles obtained were consistent with the results obtained by Marwa et al. (2013) who formulated diclofenac sodium niosomes using ether injection method. As suggested by Uchegbu and Vyas (1998), incompletely formed bilayers could be the effect of residual ethanol from the formulation process which causes an additional phase transition, thus affecting membrane rigidity. It could also be attributed to the formulation having a higher ratio of cholesterol which is known to have great impact on bilayer integrity. Crystals observed in Tween 60 formulations could be cholesterol or Tween 60; determining this conclusively will require other characterization techniques that show the interaction among various niosomal constituents like Fourier transform infrared spectroscopy (FTIR), X-ray diffraction analysis or differential scanning calorimetry (DSC) (Vankayala et al. 2018; Taymouri and Varshosaz 2016; Ruckmani and Sankar 2010).

Particle sizes contrasted with those previously obtained by Yoshioka et al. (1994) and a number of other researchers (Ghafelehbashi et al. 2019; Nowroozi et al. 2018). They had established that the surfactant with a higher HLB value and larger head group resulted in vesicles of larger sizes, with those of Tween 60 (14.7) being greater than Span 60 (4.7). Results obtained here where Span 60 formed larger sized niosomes than Tween 60 have however also been noted by Junyaprasert et al. (2012) with niosomes of ellagic acid, Bayindir and Yuksel (2010) with paclitaxel, and Ruckmani and Sankar (2010) with zidovudine. In their study with Tween 80 and Span 80, Nadzir et al. (2017) showed that lower HLB values of surfactants composition could indeed result in larger sized niosomes. Basiri et al. (2017) noted that increased Span 60 in the surfactant ratio of Span 60:Tween 60 combination increased particle size; on the contrary the formulation with the highest total quantity of surfactant as well as highest Tween 60 ratio in the experiment gave the smallest vesicles. This was said to be influenced by the larger hydrophilic head group and high HLB value of Tween 60, while the larger sizes were as a result of Span 60 being more hydrophobic and having a higher critical packing parameter (CPP) than Tween 60. Generally, higher concentrations of both components gave larger sized vesicles. Gugleva et al. (2019) made the same observations with niosomes of doxycycline, as well as Taymouri and Varshosaz (2016) with carvedilol nano-niosomes. According to Gugleva et al. (2019), increased surfactant concentration would occupy a larger membrane area together with the aryl chain leading to chain distortion, increased membrane fluidity and vesicle size.

Changes in cholesterol concentrations seemed to have more impact on particle size than changes in surfactant concentrations, as well as more effect on Span 60 formulations than Tween 60 formulations similar to observations made by Akbari et al. (2015) and Nowroozi et al. (2018). This was attributed to the hydrophilicity of Tween 60 and the increase in cholesterol being insufficient in affecting the hydrophobicity of the bilayer. Comparing formulations with combined and single surfactant(s), the effect of surfactant type can be clearly seen. For the three groups of cholesterol concentrations, all the Tween 60 containing formulations had the smallest particle sizes, the co-surfactant formulations were larger and the Span 60 only formulations had the largest sizes. This is similar to the observations made by Naderinezhad et al. (2017).

Drug loading would ideally cause an increase in particle size as depicted by most of the Span 60 formulations which is in line with results that have been reported by various authors (Kaur et al. 2016; Taymouri and Varshosaz 2016). It is due to space taken up by the added drug molecules after drug loading, although others have also recorded no change in particle size due to drug loading (Tavano et al. 2013). In contrast, a slight decrease for Tween 60 formulations was observed with K1 although exhibiting the highest EE showing a reduction in particle size. A possible reason for this is that despite pilocarpine hydrochloride being a hydrophilic drug and preferentially entrapped in the aqueous core, some of the drug could be deposited in the hydrophobic bilayer as a result of hydrophobic/hydrophobic interactions between the drug, cholesterol and surfactants as explained by Ghafelehbashi et al. (2019) in the niosomal encapsulation of cephalexin. Studies conducted by García-Manrique et al. (2020) corroborated these findings when they showed that hydrophilic drugs sometimes interact with the lipid bilayer membrane and the drug is incorporated there leading to reduced surfactant curvature and a consequent reduction in vesicle size. Similar results were reported by Tavano et al. (2013) with doxorubicin niosomes where particle size reduced after drug loading which was attributed to electrostatic attractions between the drug and bilayer causing increased vesicle cohesion, a closely packed membrane configuration and increased membrane curvature. Akbari et al. (2013) with ciprofloxacin-loaded nano-niosomes, and Lu et al. (2019) also noted the same reduction in size.

Zeta potential measures particle surface charge. The technique employed here was electrophoretic light scattering (ELS) which measures electrophoretic mobility, a function of zeta potential. Electrophoretic mobility is the velocity of particles moving through an electric field and is obtained by determining the frequency change of laser light scattered as they move (Wilson et al. 2001). Although pilocarpine HCl is a cationic drug, zeta potentials determined for all formulations regardless of cholesterol concentration, surfactant type and concentration were indicative of negatively charged vesicles. This could be due to cholesterol which inputs a negative surface charge on the vesicle as demonstrated by Farmoudeh et al. (2020) with methylene blue-loaded niosomes. They also showed that higher cholesterol concentrations resulted in increased zeta potential which supports the results obtained here. Manosroi et al. (2010) also confirmed the negative charge inducing effect of cholesterol in their study with niosomes of the cationic drug gallidermin and attributed it to uneven polarity distribution of cholesterol’s hydroxyl group. Increased surfactant ratio saw an increase in electrical conductivity. This is in accordance with determinations made by Dukhin and Goetz (2006) and Smith and Eastoe (2013) in their studies related to conductivity of surfactants in non-polar liquids.

The higher negativity of the Span 60 formulations may be attributed to ionic dissociation with resultant ionic impurities as suggested by Dukhin and Goetz (2006) and Guo et al. (2010). Results by Sadeghi et al. (2020) implicated the phosphate buffer as a contributor to the negative charge on the particles. They stated that in phosphate buffer, niosomal formulations of cationic lysozymes were surrounded by layers of ‘counter-ions’ with opposite charges to those of the niosomes. The implication of these zeta potential values is that on long term storage, the Span 60 formulations would be expected to show less tendency for aggregation, hence greater stability. This is because having higher values, there is more electrostatic repulsion and stabilization resulting in a lesser tendency for particle aggregation in the colloidal system (Seleci et al. 2016; Uchegbu and Vyas 1998). These results are supported by those obtained by Gugleva et al. (2019) where zeta potentials were negative across board, with Span 60 formulations being more negative than equivalent Tween 60 formulations.

Generally, zeta potentials of greater than − 30 mV or + 30 mV are said to be acceptable indicators of good stability (Cho et al. 2013; Khan et al. 2017). Zeta potential and associated stability could be improved particularly for the Tween 60 formulations by adding a negative charge inducer such as dicetyl phosphate (DCP) that has been widely applied in niosomal formulations and has been established to be effective in improving stability (Okore et al. 2011; Sezgin-Bayindir and Yuksel 2012; Nayak et al. 2020).

Low EE values were possibly due to the hydrophilic nature of pilocarpine hydrochloride as it is well-established that better entrapment efficiency is generally achieved with more hydrophobic drugs than hydrophilic drugs (Hashemi Dehaghi et al. 2017; Bhardwaj et al. 2020). With the hydrophilic drug being soluble in the aqueous phase, during vesicle formation the amount of aqueous phase encapsulated in the core is much less than that outside the lipid bilayer, resulting in lower percentage entrapment compared with hydrophobic drugs that have preference for the bilayer (Joshi et al. 2020). It is also known that for hydrophilic drugs, the Tween series of surfactants give the best entrapment efficiency as seen from work done by Kumar and Rajeshwarrao (2011). Tween 60 possesses a larger hydrophilic head with long alkyl chain length and hydrophilic drugs are typically entrapped in the polar aqueous core, so it enables more solubilization and entrapment of the drug (Naderinezhad et al. 2017; Manosroi et al. 2003). This was seen in work done by Ghafelehbashi et al. (2019) with cephalexin and Gugleva et al. (2019) with doxycycline where Tween 60 alone gave a higher EE compared with Span 60 alone. Span 60 on the other hand although having the same chain length (C18) as Tween 60 has a smaller hydrophilic head group so does not take up as much of the hydrophilic drug (Bhardwaj et al. 2020; Wang and Gao 2018). Manosroi et al. (2003) studied the characteristics of vesicles formed with various non-ionic surfactants and cholesterol mixtures, showing that the Tween with a C18 alkyl chain and a higher HLB value gave a better EE than the equivalent C18 Span due to a higher hydration of the polar head of the Tween. Another reason suggested by Gugleva et al. (2019) is that as the surfactant:cholesterol molar ratio increased, the cholesterol saturation limit of Span 60 was reached resulting in bilayer disruption and drug loss. For Tween 60 due to its high HLB, this saturation limit was not reached. A number of researchers have noted that the use of the most suitable surfactant and cholesterol in a 1:1 ratio was desirable in achieving the needed enhanced bilayer compactness and increased entrapment efficiency (Balakrishnan et al. 2009; Barakat et al. 2014; Bayindir and Yuksel 2010). This was, however, not the case for the Span 60 containing counterpart comparing K2 having more surfactant than K5 but EE of 21.9% and 25.1%, respectively.

Co-surfactant formulations gave better EE than those with only Span 60 and were second only to those with Tween 60 alone, demonstrating well the effect of surfactant combination. Naderinezhad et al.(2017) had similar results with Tween 60, Tween 60/Span 60 combination and Span 60 giving the highest to lowest EE of doxorubicin and curcumin in that order. In our experiments, there was, however, an exception to this trend in the group containing half the surfactant concentration, with K5 containing Span 60 alone having a higher EE than the combination but still maintaining a lower EE than Tween 60 alone. From results obtained by Barakat et al. (2014) combining a hydrophilic and hydrophobic non-ionic surfactant in niosomal formulations of hydrophilic vancomycin hydrochloride, increased EE with co-surfactant was as a result of integration into the bilayer structure by mainly hydrophobic molecular interactions of the surfactants alkyl tails as well as hydrogen bonding between the closely packed polar head groups. This resulted in increased hydrophilicity of the bilayer, hence the increased entrapment of hydrophilic vancomycin.

Cholesterol gave more stability to the bilayer, increasing rigidity and reducing permeability (as Tweens typically need cholesterol to form stable vesicles), hence more drug is retained in the vesicle (Manosroi et al. 2003). Hashemi Dehaghi et al. (2017) made similar observations with hydrophilic dorzolamide-loaded niosomes. Basiri et al. (2017) demonstrated the role of increased concentrations of cholesterol in improving EE in niosomes. It was noted to do this by increasing the chain order of bilayers in liquid state, thereby abolishing the phase transition of the system. The conclusion can be drawn thus, that in using the same surfactant and cholesterol concentration ratio in K1, it was sufficient to increase cohesion of non-polar portions in the bilayer thereby inhibiting drug leakage (Di Marzio et al. 2011). This was particularly observed in the difference in EE be Tween K1 and K7 as highlighted above. Similar results were obtained by Guinedi et al. (2005) with acetazolamide-loaded niosomes prepared by reverse-phase evaporation and thin film hydration.

### Effect of sonication on niosomal properties

It was established that indeed sonication reduced vesicle size and that longer durations produced smaller vesicle sizes. This is in line with reports on the effect of sonication observed by Sezgin-Bayindir and Yuksel (2012) who showed that optimum size reduction in their experiment was obtained after probe sonication for 60 min. For Tween 60 niosomes which had a generally lower size range, there was overall a smaller degree of size reduction than the Span 60 formulation. This can be attributed to the fact that the Tween 60 formulation had more thermodynamic stability and achieved equilibrium quickly with minimal size reduction compared to the Span 60 formulation (Diskaeva 2018). As size reduced, the PDI was also seen to reduce, indicating that the dispersion became more homogenous with sonication. Nowroozi et al. (2018) obtained similar results after bath sonication of niosomal formulations prepared with Span 60, as well as other studies investigating the effects of sonication by Akbari et al. (2013), Pereira-Lachataignerais et al.(2006), and Yeo et al. (2019).

Overall, there was more impact on the zeta potential of the Span 60 formulation than the Tween 60 formulation. This is possibly due to the occurrence of only minimal changes in size with the Tween 60 formulation, suggesting that there might be some relationship between changes in particle size and zeta potential (Shi et al. 2018). Formulation K8's behaviour was explained by Nakatuka et al. (2015) stating that smaller particles are more easily affected by surrounding particles and random fluid flow movement in a Brownian diffusion effect, hence there is easy collision with other particles. Smaller particles therefore have a relatively greater surface charge than larger particles. These results correspond to those reported when Akbari et al. (2013) that determined the effect of increased sonication durations on zeta potential, noting an increase in potential with increase in time as particle size reduced.

Ultrasonic effects on the lipid membrane result in opening and shutting of niosomes in a process of reformulation explained by Widayanti et al. (2017), so more drug is entrapped within the aqueous core with each opening. This can account for the increase in EE with K7. Decrease after 60 min is similar to what Mavaddati et al. (2015) observed and was probably due to vesicle destruction leading to drug leakage (Khan et al. 2017; Zhang et al. 2020). This trend was also observed by Anbarasan et al. (2013) with capecitabine-loaded niosomes. Considering that drug loading increased particle size for K8, the reduced EE after 30 min of sonicating could be as a result of smaller vesicles forming similar to observations by Shete et al. (2012). Some studies have shown progressive reduction in EE with longer sonication times due to reduction in particle size (Nayak et al. 2020). However, it was noted that further sonication to 60 min slightly increased EE although there was a continued decrease in size. A possible explanation for this is that along with the dispersion gaining thermodynamic stability, the bath sonication process also facilitates hydration by the aqueous buffer containing pilocarpine HCl so encourages drug entrapment (Mavaddati et al. 2015). A similar effect on EE attributed to increased hydration time was noted by (Yeo et al. 2019).

The two formulations were observed to exhibit opposite trends; the K7 increasing after 30 min and then decreasing after 60 min, and K8 the reverse. A possible reason for this can be tied to the characteristics of the surfactants, with Span 60 known to form a more cohesive/stable lipid membrane with cholesterol due to its hydrophobicity compared to Tween 60, hence would be less likely to be destroyed under the same conditions as Tween 60 formed membrane (Bagheri et al. 2014).

## Conclusion

Tween 60 formulations gave more homogenous dispersions, more desirable particle sizes for ocular delivery and better EE than Span 60 formulations. Sonication was seen to reduce particle size, improve PDI and increase zeta potential by approximately 28% and EE by 61%, with optimum sonication time for this study pegged at 30 min. These results are specific to the conditions used in this experiment and cannot be exhaustively and broadly generalized as there were no replicates. They, however, suggest that in addition to carefully selecting and varying surfactant types and concentrations, it is possible to use sonication time as a process parameter for formulating optimized niosomes of hydrophilic drugs with properties that indicate homogeneity, a low propensity for aggregation and high entrapment efficiency. Further work would be required to exhaustively determine the influence surfactant type has on the effect of sonication on entrapment efficiency. This could be useful in overcoming stability challenges and maximally exploiting niosomes advantages of greater permeability, longer ocular retention, and drug protection from metabolic degradation over conventional formulations and systems for pilocarpine hydrochloride delivery to the eye.

## Data Availability

Available upon request.
